# Royal Edinburgh Hospital for Sick Children

**Published:** 1904-05-07

**Authors:** 


### ROYAL EDINBURGH HOSPITAL FOR SICK CHILDREN.

We are requested to state that there is a printer's error in "Burdett's Hospitals and Charities" on page 201, where the average cost per bed is given as £67¼ instead of £87¼ as it should be. This error led to a statement on page 186 of "Burdett's Hospitals and Charities" for 1904 that there had been a large increase in the cost per occupied bed over that of last year for which the editor could not account. The corrected figures in the average cost per occupied bed are in 1901 £87¼, and in 1902 £89, showing an increase for the year of £1 15s. per occupied bed. The editor much regrets this error, which he asks us to correct, and expresses the hope that subscribers to "Hospitals and Charities" for 1904 will note the correction in their copies of this year's book. The management of the Royal Hospital for Sick Children, Edinburgh, has the reputation of being both intelligent and careful, and we have great pleasure in giving insertion to this correction.

